# Assessment of the frequency of *Mycobacterium bovis* shedding in the faeces of naturally and experimentally TB infected cattle

**DOI:** 10.1111/jam.15677

**Published:** 2022-07-08

**Authors:** Si Palmer, Gareth A. Williams, Colm Brady, Eoin Ryan, Karolina Malczewska, Tim J. Bull, Philip J. Hogarth, Jason Sawyer

**Affiliations:** ^1^ Department of Bacteriology Animal and Plant Health Agency (Weybridge) Addlestone UK; ^2^ Department of Agriculture Food and the Marine (DAFM) Celbridge Ireland; ^3^ Official Veterinarian Eville and Jones Ltd, Thorpe Park Gardens Leeds UK; ^4^ St. George's University of London London UK

**Keywords:** cattle, faecal samples, limits of detection, *Mycobacterium bovis*, prevalence, shedding, TB reactors

## Abstract

**Aims:**

To assess the prevalence of *Mycobacterium bovis* bacilli in faecal samples of tuberculous cattle, and to better understand the risk of environmental dissemination of bovine tuberculosis (TB) through the spreading of manure or slurry.

**Methods and Results:**

Faecal samples were collected from 72 naturally infected cattle with visible lesions of TB that had reacted to the tuberculin skin test and 12 cattle experimentally infected with *M. bovis*. These were examined by microbial culture and PCR to assess the presence of *M. bovis* bacilli. There were no positive cultures from any naturally infected test reactor animal. A single *M. bovis* colony was cultured from a faecal sample from one of the experimentally infected animals. A single PCR positive result was obtained from the faecal sample of one naturally infected test reactor.

**Conclusions:**

The prevalence of *M. bovis* in the faecal samples of TB‐infected cattle was extremely low.

**Significance and Impact of the Study:**

The results suggest that the risk of spreading TB through the use of slurry or manure as an agricultural fertilizer is lower than that suggested in some historical literature. The results could inform a reconsideration of current risk assessments and guidelines on the disposal of manure and slurry from TB‐infected herds.

## INTRODUCTION

The potential role of slurry and manure in the persistence and spread of bovine TB has been highlighted by the recent independent review of the bovine TB strategy for England (Godfray et al., 2018). If cattle faeces do contain significant amounts of viable *Mycobacterium bovis (M. bovis)* bacilli (the causative agent of bovine TB), then the common agricultural practice of manure and slurry spreading may represent a significant risk pathway leading to environmental contamination, and thus a potential route of TB infection for grazing livestock and wildlife. *Mycobacterium bovis* is known to be able to survive in the environment for considerable amounts of time, which could constitute a potential reservoir of infection. There is renewed interest and research exploring the possible role of such environmental contamination in both the persistence and transmission of bovine TB (reviewed by Allen et al., 2021).

The risk posed by cattle faeces in the spread of TB has been reviewed (de la Rua‐Domenech, 2007; McCallan et al., 2014). However, there are limited published data on the prevalence and load of *M. bovis* in cattle faeces. These data are contradictory, or many decades old when disease burden, cattle testing regimes and *M. bovis* detection methods were different. Some of these studies did, however, suggest significant levels of *M. bovis* in faeces sampled from TB test reactor cattle (Reuss, 1955) and cattle experimentally infected with *M. bovis* (Neill et al., 1988). Because of improved TB surveillance regimes for cattle herds, most infected cattle in the UK and Ireland are detected at an early stage of infection (with decreased disease‐associated pathology and fewer tuberculous lesions), which may explain why some older publications reported high levels of shedding (reviewed by McCallan et al., 2014). Indeed, observation of clinical TB in cattle is now a very rare occurrence in those countries.

The routes of shedding of *M. bovis* into faeces, and to what extent shedding occurs in TB infected cattle, is poorly understood. It could be speculated that shedding into faeces is most likely when animals have TB lesions in mesenteric lymph nodes associated with intestinal infection. However, this would require the lesions to have undergone significant caseous necrosis and subsequent cavitation into the gut lumen. Although a study of pathology in TB reactor cattle in the UK (Liebana et al., 2008) did note that caseous necrosis was common in TB reactors, cavitation was not noted in this study. Lesions in TB test reactors are most common in the lymph nodes of the thoracic cavity (bronchial, mediastinal) and in the retropharyngeal lymph nodes, but lesions in the mesenteric lymph nodes do occur in naturally infected TB reactor cattle. Liebana et al., 2008 found that about 10% of TB test reactors in the UK had lesions of TB in those lymph nodes. Finally, as a potential route of *M. bovis* shedding into the gut, if cattle with respiratory tract lesions cough and subsequently swallow *M. bovis*, it is theoretically possible that a proportion of these bacteria may survive and remain present in the faeces excreted by cattle.

In short, it is not clear what levels of *M. bovis* are present in the faeces of TB infected cattle and what, if any, are the main routes of shedding. This in turn makes it difficult to assess the role that cattle manure and slurry play in the spread and persistence of *M. bovis* and hence bovine TB.

This study set out to obtain data on the frequency and bacterial load of *M. bovis* in the faeces of naturally and experimentally TB infected cattle in the UK and Ireland, to inform risk assessments and potentially direct future research in this area. Faecal samples were examined by microbial culture and PCR with the aim of detecting any *M. bovis* present in the samples. Two culture methods were used—an established *M. bovis* faecal culture method and a novel mycobacterial culture method called TiKa(Bull et al. 2016). The PCR method was a qPCR employing a *M. bovis*‐specific assay and a 16S DNA assay, which allowed the quality of the extracted DNA and the presence of PCR inhibitors to be checked.

## MATERIALS AND METHODS

### Collection of faecal samples

Faecal samples were taken at slaughter from 42 cattle that had reacted to the single intradermal comparative cervical tuberculin (SICCT) test and presented with visible lesions (VL) typical of TB at post‐mortem examination. Sampling took place in February and March 2020 during five separate visits to a slaughterhouse in the west of England approved for slaughter of TB test reactor cattle. Animals were identified as VL reactors by the duty Official Veterinarian, which allowed subsequent collection of faecal samples specifically from these animals when the intestines were removed later in the slaughter process. These animals were submitted from 29 different farms. For each VL reactor, two separate faecal samples (approximately 50 g) were collected from different areas in the rectum as close as possible to the anus. For one animal, eight additional separate 50 g samples were taken from different locations in the rectum and lower large intestine to test the hypothesis that distribution of *M. bovis* may be uneven in this region of the gut. Samples were transported to the laboratory at ambient temperature.

Faecal samples were also collected from 30 skin test reactor cattle housed at a research facility in Ireland by the Department of Agriculture, Food and the Marine (DAFM). These animals had been brought to the facility after reacting to the SICCT test on their respective farms and had been subsequently tested using an interferon gamma release assay to confirm their TB infection status. One single faecal sample (approximately 20 g) was collected from the rectum of each live animal using a lubricated gloved hand. Samples were transported to the laboratory at ambient temperature. At the time of writing, 16 of the 30 animals had subsequently been subjected to post mortem examination.

Faecal samples (approximately 20 g) were also taken from 12 individual cattle experimentally infected with *M. bovis* as part of two different and separate experiments being carried out at the Animal and Plant Health Agency (APHA) Weybridge (four animals from experiment 1 and eight animals from experiment 2). In all cases, male calves (between 4 and 7 months of age) were sourced from officially bovine TB free herds in GB that had no recent history of infection and were housed at APHA Weybridge throughout the experiment. Calves were experimentally infected with a virulent strain of *M. bovis* (AF2122/97) via the endobronchial route as previously described (Villarreal‐Ramos et al., 2018), with the exception that the infectious dose was delivered just below the main bifurcation between the left and right lungs. The infection inoculum was cultured to determine the *M. bovis* CFUs (colony forming units) delivered (5200 CFU for experiment 1; 5660 CFU for experiment 2). Faecal samples (approximately 20 g) were collected weekly post infection from live animals by insertion of a gloved finger into the anus to induce defecation. Experiment 1 had 14 sampling dates (one prior to infection, thirteen post infection), and Experiment 2 had nine sampling dates (all post infection). Post‐mortem examinations were performed on the infected animals at the end of the experiment (9 to 13 weeks post infection) to assess TB associated disease pathology in the lungs and lymph nodes. All calves showed visible TB pathology, which was confirmed by the culture of *M. bovis* from post‐mortem tissue samples. All animal procedures at APHA were approved by the APHA Animal Welfare and Ethical Review Board (references PF7D840A5–2‐004v2 and PF7D840A5–2‐005v1).

### Established TB culture method

In the laboratory, each 50 g faecal sample taken from the UK VL reactors had two replicate 2 g samples taken from it for standard culture. Twenty mL of 0.75% (w/v) cetylpyridinium chloride (1‐hexadecylpyridin‐1‐ium chloride; CPC) was added to 2 g of faeces and, after vigorous mixing, samples were left to stand at room temperature for 30 min. 10 ml of the resulting supernatant was transferred to a polypropylene tube and left overnight at room temperature. The sample was then centrifuged at 3000 X *g* for 15 min and the resulting sedimented material was re‐suspended in 2 ml of phosphate buffered saline (PBS;150 mmol I^−1^ NaCl, 9 mmol I^−1^ Na_2_HPO_4_, and 1.3 mmol I^−1^ NaH_2_PO_4;_ pH 7.6) to form the culture inoculum. 200 μl of this was inoculated onto one LJP (Lowenstein Jensen medium with pyruvate) media slope and three modified Middlebrook 7H11 (Gallagher & Horwill, 1977) media slopes, using 200 μl of inoculum for each slope. Inoculated slopes were incubated at 37°C for up to 12 weeks. For liquid culture, 500 μl of inoculum was added to a single BBL™ MGIT™ Mycobacteria Growth Indicator Tube (7 ml) to which had been added 0.8 ml of BACTEC™ MGIT™ 960 growth supplement containing BD BBL MGIT PANTA antibiotic mixture (prepared according to manufacturer's instructions, all tubes and reagents Becton Dickinson, Wokingham, UK). Tubes were incubated and monitored in a MGIT 960 instrument (Becton Dickinson) for up to 42 days. 100 μl aliquots of the inoculum were also stored at −20°C for future PCR analysis. In addition, one animal had an additional seven 2 g samples taken from one of the 50 g samples which were processed as additional samples as above (to test the hypothesis that the distribution of *M. bovis* may be uneven even within a single sample of faecal material).

For the samples taken from Irish TB reactors, each 20 g sample collected from each animal had four replicate 2 g sub‐samples taken in the laboratory for standard culture. For each faecal sample collected from experimentally infected cattle two replicate 2 g samples were taken for standard culture from faecal samples collected at each time point. Samples were processed as for the UK reactors (including storage of inoculum for PCR analysis) with the exception that the LJP slope was omitted.

*Mycobacterium bovis* positive control faecal samples were prepared by spiking faecal samples (obtained from cattle housed at APHA but not associated with TB studies) with *M. bovis,* and then processing these alongside each batch of collected study faecal samples to check successful functioning of reagents and media. The preparation of spiked material is described in the section titled “Determination of the limit of detection of culture and PCR methods”.

### TiKa culture method

For each 2 g sample taken for standard culture, a 2 g sample was also taken for TiKa culture. The faecal sample was resuspended in 5 ml of PBS (as above). The resulting suspension was filtered using a BagPage® 100 sterile filter bag (Interscience, Saint Nom la Brétèche, France). The resulting filtrate was centrifuged at 1500 × *g* for 10 min at room temperature. The resulting pellet was resuspended in 5 ml of fresh PBS and 150 μl of the resulting suspension was added to 10 ml of TiKa‐Kic solution (TiKa Diagnostics Ltd, London, UK) in a 30 ml sterile glass universal tube. TiKa‐Kic solution was prepared by adding TiKa supplement T1 to TiKa‐Kic buffer at a ratio of 1 μl per mL respectively. Samples were incubated overnight (18 h) at 37°C with rotational shaking at 160 rpm. The samples were then centrifuged at 1500 × *g* for 10 min at room temperature. The supernatant was discarded and sedimented material was resuspended in 600 μl of media taken aseptically from a BBL™ MGIT™ Mycobacteria Growth Indicator Tube (7 ml). This was added back into the growth indicator tube along with 0.8 ml of BACTEC™ MGIT™ 960 growth supplement containing BD BBL MGIT PANTA Antibiotic Mixture (BD as previous) and 8.5 μl of TiKa‐B1 supplement (TiKa Diagnostics Ltd). Tubes were incubated and monitored in a MGIT 960 instrument (BD) for up to 42 days. Culture from any MGIT™ tubes that indicated bacterial growth were checked by Kinyoun cold stain microscopy (as described below) and, if acid‐fast organisms were observed, then PCR was used to confirm the presence of *M. bovis* using a RD4 real‐time PCR as described below.

### Kinyoun cold stain and microscopy for detection of acid alcohol‐fast bacilli (AAFB)

Approximately 100 μl of sedimented material from each MGIT™ Mycobacteria Growth Indicator Tube was transferred to a glass microscope slides using a sterile pastette and allowed to dry on a slide dryer/heater. Slides were then submerged in methanol for 30 min and allowed to air‐dry before heat fixing for 5 min using a slide heater. Slides were then covered with Kinyoun carbol fuchsin (Pro‐Lab Diagnostics, Birkenhead, UK) and left for 15 min. Following rinsing with tap water, slides were flooded with Kinyoun differentiator (Pro‐Lab Diagnostics) for 10 min, applying a differentiator solution change after 5 min. Following rinsing with tap water, the slides were counterstained with 1% methylene blue or 1% malachite green (both Pro‐Lab Diagnostics) for 1–3 mins. Then, the slides were rinsed well with tap water and then examined microscopically using an oil immersion objective.

### DNA extraction from culture inoculum prepared from faecal samples

Prior to removal from the Containment Level 3 laboratory, 100 μl aliquots of frozen culture inoculum stored for PCR analysis were defrosted and heat inactivated in a Techne Dri‐Block® (Cole‐Palmer, Stone, UK) at 80°C for 1 h and then refrozen at −20°C before PCR analysis at Containment Level 2.

All microbiology culture inocula from UK and Irish reactor faecal samples were taken forward for subsequent DNA extraction and PCR. For experimentally infected animals, inoculum samples for PCR were limited to the last sampling points prior to euthanasia and post‐mortem: for Experiment 1, samples taken at 11, 12 and 13 weeks post infection; for Experiment 2, samples taken 7 and 8 weeks post infection.

DNA extraction was carried out using the MagMAX Core kit (ThermoFisher, Loughborough, UK). 100 μl of heat‐treated inoculum resulting from the standard culture procedure as described above was added to 1 ml of lysis‐binding buffer (from the MagMAX Core kit) in a MP Biochemicals Lysing Matrix Lysis B tube (VWR Ltd, Lutterworth, UK). These were then disrupted in a Precellys 24 homogenizer (Bertin Instruments, Montigny‐le‐Bretonneux, France) at 6000 rpm for 30 s. Following centrifugation for 1 min at 16500 X *g*, 800 μl of the resulting homogenate (avoiding glass beads and any residual debris) was removed from the Lysing Matrix tube and then purified using a Kingfisher FlexPlus magnetic particle processor (ThermoFisher) and the MagMAX Core kit using the “Core kit no heat protocol” with an elution volume of 90 μl following the manufacturer's kit instructions.

Heat treated culture inoculum prepared from spiked faecal samples were run as extraction positive controls alongside extraction negative controls during DNA extraction.

### Spoligotyping

DNA prepared from isolated cultures or liquid culture was spoligotyped following the reverse line blotting technique (Kamerbeek et al., 1997). Spoligotyping membranes were manufactured at APHA. Spoligotypes were named following the naming conventions outlined in Smith and Upton (2012).

### Real‐time PCR

Real‐time PCRs were prepared using TaqMan® Environmental Master Mix (ThermoFisher) and dispensed into 20 μl aliquots to which 5 μl of DNA was added. Concentrations in the final PCR were: 2.5 mmol I^−1^ MgCl_2_, 1 × master mix, 0.4 μmol I^−1^ forward and reverse primers, 0.1 μmol I^−1^ dual‐labelled probe labelled at the 5' end with FAM (6‐carboxyfluorescein) and at the 3' end with BHQ‐1 Dark Quencher. Samples were amplified with a universal bacterial 16S assay (Wakeley et al., 2006) to check that DNA was capable of being PCR‐amplified. Primer and probe sequences of the universal assay were Univ F primer 5' ACTACGTGCCAGCAGCC 3', Univ primer R 5'GGACTACCAGGGTATCTAATCC 3', Univ probe 5’ TGTTTGCTCCCCACGCTTTCGCAC 3'. Samples were also PCR amplified with an RD4 PCR assay specific for *M. bovis* (Sweeney et al., 2007) with the following sequences: *M. bovis* F RD4 primer 5'TGTGAATTCATACAAGCCGTAGTCG3', *M. bovis* R RD4 primer 5'CCCGTAGCGTTACTGAGAAATTGC3', *M. bovis* RD4 probe 5'AGCGCAACACTCTTGGAGTGGCCTAC 3'. All primers and probes were from Sigma‐Aldrich (Gillingham, UK). Reactions were cycled on an AriaMX PCR instrument (Agilent Ltd, Wokingham, UK) as follows: 95°C for 10 min; 40 cycles of 95°C for 15 s, 58°C for 1 min. C_T_ (cross threshold) values were determined by the AriaMX software (version 5.1) selecting the ΔRn fluorescence and curve smoothing analysis options. PCR positive and negative controls were included during each PCR run.

### Determination of the limit of detection of culture and PCR methods

Limits of detection for the methods used in this study were determined by spiking faecal samples from TB negative cattle with known quantities of *M. bovis* and then testing the resulting samples using the culture and PCR methods described above. The *M. bovis* (strain AF2122/97) was enumerated before and after spiking, by plating onto modified Middlebrook 7H11 plates, a serial dilution of the culture to ensure consistency and accuracy in the estimated numbers of bacteria. Spiking was used to produce 2 g faecal samples containing a range of concentrations from approximately 10^6^ to 10^1^ CFU *M. bovis*. For the established culture method, the limit of detection was determined by growth on media plates (using the same media, modified Middlebrook 7H11) to aid visualization and enumeration of recovered bacterial colonies and was based on results from three separate replicate experimentsFor TiKa culture, spiked samples were monitored for growth in the MGIT instrument and subsequently tested using the RD4 real‐time PCR to confirm the presence of *M. bovis*. For the PCR method, following production of culture inoculum from the spiked samples and DNA extraction, detection was based on recording of a C_T_ value less than 40 cycles in the real time PCR (the number of cycles run in the PCR). Spiked samples for PCR were also subjected to the same freeze thaw and heat block procedure to mimic the treatment of test samples.

**FIGURE 1:**
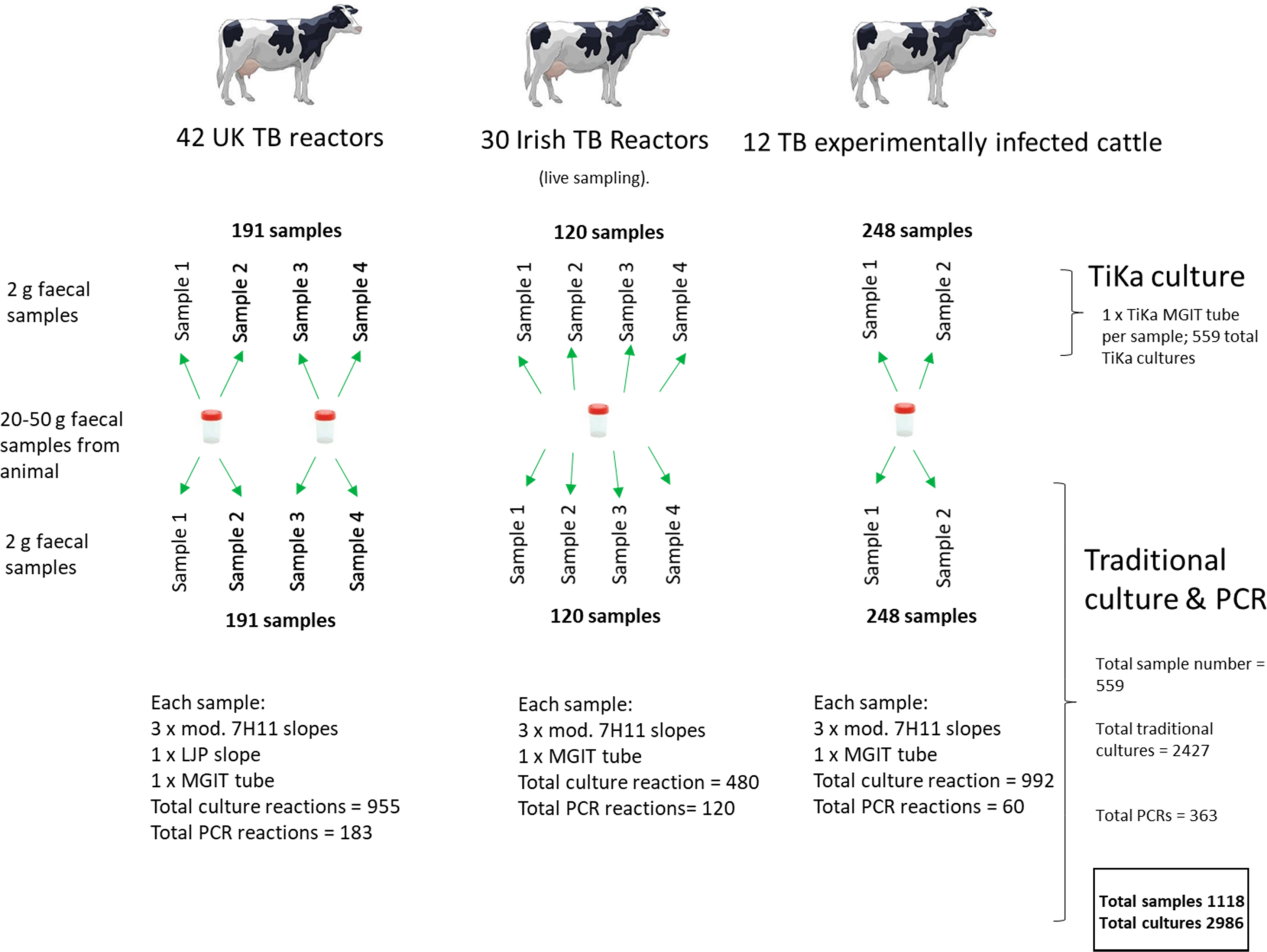
Sampling and testing scheme used in the study. For UK TB reactors, 191 faecal samples in total for traditional culture and PCR. Two replicates of two duplicate samples from 42 animals (168 × 2 g samples) plus 7 additional samples from one 50 g pot from one animal; plus 16 extra replicates (2 × 2 g) from 8 × 50 g samples taken from different positions in rectum. For PCR, only half of these 16 extra replicate were tested; total 183 PCRs. Additional 191 samples for TiKa culture. For Irish TB reactors, 4 × replicate 2 g samples taken from a single faecal sample collected from 30 animals (120 total). 120 additional samples taken for TiKa culture. For experimentally infected animals, 248 samples in total; 124 individual faecal samples taken from 2 separate experimental infection experiments (Experiments 1, 4 animals for 13 time points; Experiments 2, 8 animals for 9 time points) tested in duplicate. PCR samples were limited to the last time points prior to post mortem: For Exp.1 samples taken at 11, 12 and 13 weeks post infection (12 samples); for Exp. 2 samples taken at 7 and 8 weeks post infection (18 samples); 30 samples total in duplicate, total 60 samples.

The sampling regime, test methods and test numbers for this study are summarized in Figure 1.

## RESULTS

*Mycobacterium bovis* was not cultured from the faecal samples collected from any of the UK or Irish TB reactor (naturally infected) cattle. A single *M. bovis* colony (confirmed by spoligotyping to be the same strain used for experimental infection) was detected on a single media slope for one of the experimentally infected animals. A single PCR positive sample was detected from an Irish reactor animal, out of the four samples tested by PCR from that animal. Subsequent PCR testing of another duplicate set of stored samples from this animal yielded negative results.

The standard culture method was estimated to have had a mean limit of detection of 211 CFU (range 125–312)/2 g faecal sample (or approximately equivalent to 100 CFU/g faeces). The proportion of *M. bovis* present in a 2 g faecal samples that could be cultured using the standard culture method was estimated to be 2.1%–5.3%. The limit of detection of TiKa culture was slightly lower than that of standard culture, at approximately 10 CFU/g faeces indicating that it is perhaps a more sensitive *M. bovis* culture method for faecal samples. The limit of detection of the PCR method was higher than the culture‐based methods, at approximately 10^5^ CFU/g faecal sample.

The contamination rate for the standard method solid culture was ~15–20% for Modified Middlebrook 7H11 but higher for Lowenstein Jensen medium (up to 40%). The contamination rate for the TiKa protocol used in this study was ~16% of samples.

The use of the universal bacterial 16S assay demonstrated that DNA was amplifiable with C_T_ values ranging from 25 to 35.

Of the 42 UK VL reactor cattle, 22 (~52%) were recorded with lesions in bronchial and mediastinal lymph nodes, with 13 (~31%) in retropharyngeal nodes. There were also examples of lesions in parotid (3), mesenteric (2) and hepatic (1) lymph nodes. Two animals displayed lesions in the liver. All lesions were described as “typical TB lesions” with all but 2 animals displaying multiple lesions. The majority of lesions were in the 2–10 mm size bracket (29, ~69%) with 11 animals (26%) displaying larger lesions in the 11–50 mm category. Two animals displayed small (<2 mm) multiple lesions. Lesions in the majority of animals (38, ~90%) were classified as caseous, with 4 classified as calcified and 2 as caseous and purulent. The 16 Irish reactors subjected to post‐mortem showed 14 with visible lesions and 2 with no visible lesions. Those with lesions had a similar distribution of lesions to the UK reactors with the most being present in the lymph nodes associated with the head and chest with two animals displaying lesions associated with the liver. There was a single mesenteric lesion noted. The experimentally infected cattle were subjected to examination and culture of respiratory associated thoracic lymph nodes (cranial mediastinal, caudal mediastinal, right and left bronchial, cranial tracheobronchial), as well as assessing samples from all 8 lobes of the lung. In addition, the carcasses were examined for signs of lesions elsewhere. All experimental infected animals exhibited multiple lesions and produced subsequent *M. bovis* culture results from these lymph nodes and lung samples. There was one example of a liver lesion in a single animal. The single animal that produced the positive culture result had visible gross lesions in all but one of the thoracic lymph nodes examined, and in 2 of the 8 lung lobes and was ranked 4 out of 8 in terms of overall TB pathology from the 8 animals that were part of the same infection experiment.

In the absence of any replicated positive results within individual faecal samples or cattle, the results can be crudely interpreted by assuming that all outcomes were independent. There was one positive culture out of a total of 2986 grown from faecal samples taken from TB infected cattle in this study. Assuming all infected cattle are equivalent, the observation gives 95% confidence that fewer than 1/630 cultures from infected cattle will be positive.

## DISCUSSION

In total, over 2986 individual cultures (2427 conventional culture methods, including media slopes and liquid culture and 559 TiKa cultures) and 363 PCRs were conducted to assess the frequency of *M. bovis* shedding in the faeces of 72 naturally infected (TB reactor) and 12 experimentally infected cattle.

The single *M. bovis* colony detected and single *M. bovis* PCR positive result recorded in this study suggest that shedding of *M. bovis* in the faeces of TB infected cattle (both naturally and experimentally) is rare or, if it happens, the bacterium is generally present at levels below 10–100 *M. bovis* /gram of faeces (the approximate limit of detection range of the culture methods used). However, the single positive PCR result suggest that *M. bovis* was present at higher levels (given the limit of detection of the PCR is 10^5^ *M. bovis*/g faeces) in very localized areas within the faeces of the individual animal which produced the positive test result (given the other replicate and repeat samples from this animal tested negative). In these circumstances, then these bacilli could subsequently contribute to the overall bacterial load in slurry and manure.

It is not possible to completely rule out the possibility that the single positive culture and PCR result were caused by contamination during the testing process. However, steps and controls were applied to mitigate against this possibility. Positive control cultures were handled and cultures set up (in biological safety cabinets) only after the completion of processing batches of test samples. PCR reactions included PCR DNA negative extraction controls and PCR negative controls.

These results show that shedding of large amounts of bacteria in faeces of infected cattle in a similar way to *Mycobacterium avium* subsp. *paratuberculosis* (MAP) in Johne's disease (up to 10^7^ organisms per gram of faeces; Pradhan et al., 2011) does not commonly occur in cattle infected with TB. These findings correlate with the known significant differences in the pathogenesis of these two genetically related organisms, with MAP infection predominantly resulting in a chronic enteritis and *M. bovis* being largely an intracellular pathogen of macrophages in the respiratory tract and associated lymph nodes.

It is not surprising that some evidence of the presence of *M. bovis* in faecal samples from TB infected animals was found. Older literature has reported the presence of *M. bovis* in the faeces of TB infected cattle based on culture (Maddock, 1936; Neil, 1988; Reuss, 1955), although more recent published information on the levels of *M. bovis* in the faeces of current TB reactors is not evident in the literature (McCallan et al., 2014). There are also reports of *M. bovis* being detected in the faeces of animals such as badgers (Gallagher & Clifton‐Hadley, 2000; King et al., 2015) and foxes (Richomme et al., 2020), although recent studies rely on PCR as the method of detection. A recent study using PCR as a tool to identify infected badger social groups did report *M. bovis* positive faecal samples taken from badger latrines (King et al., 2015). However, this approach required multiple samples to be collected/tested per latrine and positive samples were relatively uncommon, even from samples collected from latrines known to be used by *M. bovis* positive animals. Routine culturing of badger faecal samples from a long‐term study site in Woodchester Park, Gloucestershire, with confirmed TB positive animals, resulted in low numbers of TB positive cultures (Delahay et al., 2013). Again, this suggests that although shedding of *M. bovis* in faeces can occur during TB disease progression, routine detection can be challenging and often require significant sampling and testing effort.

Shedding of *M. bovis* in faeces is more likely in advanced TB disease progression when infection has disseminated throughout the body. Due to the extremely rare observation of clinical signs of TB in UK and Irish cattle, it can be surmised that the bovine TB surveillance programme in the UK and Ireland identifies animals early in the infection process, well before disseminated disease has occurred. In contrast, the lack of routine TB surveillance program for the wildlife species (noted above) may be leading to individual animals having more advanced disease and hence being more likely to shed *M. bovis*.

Even if the level of shedding of M. bovis in faeces is very low or shedding is a rare event, the large volumes of cattle slurry generated on some farms may mean this is still a potential source of infection

Given the limits of detection of the methods used in this study, it is possible that *M. bovis* is present at sub‐detectable levels in the faeces from reactor cattle. The large volumes of faeces spread on farmland (via slurry and manure) may mean that even very low levels of *M. bovis* could represent a risk of spread of disease, although it is difficult at present to quantify this risk. Alternatively, relatively rare individual shedding events, not detected in this study, could be contributing to the spread of the disease at a population level.

However, despite the widely accepted view that spreading of slurry and manure represents a risk for TB spread (and hence development of precautionary guidelines on storage and use of these materials), conclusive evidence of spread of TB via contamination of pasture through manure and slurry is missing from the literature (Allen et al., 2021).

Routine detection of such low levels (or definitive proof of the absence of) of *M. bovis* in faeces, or detecting rare events, is technically challenging. Obtaining such data would require significant sampling and development of improved *M. bovis* detection methods (with lower limits of detection/higher analytical sensitivity). The starting quantity of 2 g of faeces analysed in this study was a limitation on the limit of detection of the culture techniques used and obviously represents a very small sample weight considering a cow may produce tens of kilograms of faeces every day. However, the practical reality of processing large numbers of samples and larger weights for culture would make routine laboratory processing challenging and expensive.

Did the project target the right population of animals?

Our assumption in the design of this project was that the animals most likely to shed *M. bovis* in their faeces were skin test reactors with visible lesions of TB, as it is generally assumed that such animals have progressed to a more advanced stage of infection (Neill et al., 2001) and hence would be more likely to be shedding *M. bovis* in their faeces if this occurs. However, it remains possible that shedding occurs before the development of tuberculous lesions. For the faecal samples collected in the UK, VL reactor animals were specifically targeted. Of the 42 animals from which samples were taken, 24 had tissue samples submitted for culture (only samples from premises with new breakdowns are routinely submitted for culture); of these, 22 were subsequently confirmed as *M. bovis* positive by culture at APHA. These results are consistent with the high levels of recovery of *M. bovis* in bacteriological culture from TB test reactors with visible lesions.

Faecal samples were collected from TB reactors in Ireland prior to slaughter and the cattle sampled were subsequently shown to contain a mixture of VL and non‐VL animals. To date sixteen of the animals have been subjected to post‐mortem examination, fourteen of which had visible lesions and two had no visible lesions. Post‐mortem results from the other animals will be available in due course but were not available in time for publication of this study, including the one animal that produced the PCR positive result.

Although experimentally infected animals are not necessarily accurately representative of naturally infected cattle detected through field testing, they do represent another category of infected animal. The high infection dose used during experimental infection does lead to predominantly VL animals at post‐mortem (Villarreal‐Ramos et al., 2018) and all experimentally infected animals in this study were confirmed as *M. bovis* culture positive from lymph tissue taken at post‐mortem.

So, in this study a range of animals of likely different disease severity and types were targeted, with very little evidence of the presence of *M. bovis* in faeces of naturally or experimentally infected animals.

The most likely source and route of any *M. bovis* shedding in cattle faeces is poorly understood. Typical TB lesions seen in the head and chest lymph nodes associated with the respiratory tract (such as retropharyngeal and bronchial) may be a potential source of the bacterium, through swallowing of associated mucus into the digestive tract. Alternatively, mesenteric lymph nodes of TB infected cattle may be a more likely source of *M. bovis* in faeces. Thoracic TB lesions are more common, but mesenteric lymph tissue lesions do occur (Liebana et al., 2008). If such lesions do potentially offer a greater risk of *M. bovis* in faeces, then this study did not specifically target such animals. However, as noted previously, this would require the lesions to have undergone significant caseous necrosis and subsequent invasion of the gut mucosa.

In practice, it would be difficult to specifically target and sample such animals without a significantly greater sampling effort as only two of the 42 UK VL reactors from which samples were taken in this study were observed to have mesenteric lesions by the OV. Although no *M. bovis* was detected in the faeces of these animals in this study, the small sample size means it is not possible to draw any firm conclusions about the contribution, if any, of mesenteric lymph node lesions to the shedding of *M. bovis* in faeces.

More generally, UK animals with visible lesions of TB were less common than anticipated, with the result that a greater sampling effort was required than anticipated to collect the target number of VL reactors. Approximately 25% (42/165) of the TB reactor cattle presented for slaughter during the sampling visits to the UK slaughterhouse had visible lesions. This again may be the result of current TB control policies identifying cattle earlier after infection. This may explain why some historical studies identified significant levels of *M. bovis* in faeces of TB reactor cattle, but levels appear to be lower in contemporary reactors.

Did technical limitations in the methods used to detect M. bovis compromise this study?

This study was designed as an initial investigation of the presence of *M. bovis* in the faecal samples of TB reactor cattle. There are potential technical limitations of this study which should be considered when interpreting the results.

The limited scope of this study meant that it was not possible to devote a significant amount of time to *M. bovis* culture method and PCR assay evaluation and optimization—the methods used were based on, or adapted from, existing methods and protocols.

The established culture method was kindly supplied by Dr Kevin Kenny of the Central Veterinary Research Laboratory, DAFM, Ireland. The method uses CPC as the decontaminant treatment, which was largely effective in decreasing the levels of contaminating non‐mycobacterial species present in faecal samples, but some contamination of cultures remains inevitable (see below). CPC is known to be a stringent decontaminant, which also impacts on *M. bovis* survival, with the data from this study showing that only about 2–5% of the *M. bovis* bacilli (spiked into the faecal samples used to determine the limits of detection) surviving the CPC and physical treatment processes. This level of survival is based on the one strain used for producing the spiked samples. This is a strain used extensively at APHA for experimental infections and was originally isolated from a real infection and hence is a field strain. However, it has subsequently been repeatedly sub‐cultured in the laboratory. It is possible that any *M. bovis* in current field reactors is even more sensitive to the effects of CPC than the strain used in the limit of detection work reported here and that the faecal samples negative results seen in this study are due to this. The balance between using a decontamination regime that removes sufficient background flora to prevent excessive contamination while at the same time not killing the target *M. bovis* bacilli is an ever‐present challenge in culturing slow‐growing mycobacteria, which is exacerbated when attempting to culture from faecal samples. It is possible that the use of alternative decontamination agents (oxalic acid, for example) or a shorter exposure time to CPC may have produced different results.

In addition, it is possible that field strains isolated directly from faecal samples are less adapted to growth on standard artificial media and the resulting low levels of detection by culture are, at least in part, related to this. The use of the TiKa method, which uses a biological peptide‐based decontaminant, was an attempt to overcome the potential issues associated with the use of CPC and potential limitations of traditional culture approaches (Bull et al., 2016). The TiKa method has been recently used to improve the sensitivity of detection of *M. bovis* from tissue samples in wildlife species (Goosen et al., 2022). However, the negative culture results seen from TB reactor faecal samples using the TiKa method in this study also suggest that levels of *M. bovis* are low. The TiKa culture protocol employed was a first attempt at routine use of this method for *M. bovis* detection in cattle faeces. There is scope for further refinement and improvement of the technique. This is likely to involve the development of protocols capable of dealing with a higher starting amount of faeces than 2 g and through further improvement of the TiKa Kic decontamination process to further reduce the levels of contamination.

Testing of faecal samples is always a challenge given the nature of the sample and the very high levels of background flora and potential contaminants/inhibitors in this sample type. This is illustrated by the challenge to control contamination. As described in the results, despite steps to control contamination, the contamination rate for the standard method solid culture was ~15–20% for Modified Middlebrook 7H11 but higher for Lowenstein Jensen medium (up to 40%) probably due to the antibiotic/antimicrobial mix used in the former making it more suitable for use when culturing from faecal samples. There was no discernible pattern or type of sample associated with contamination and a potential way to improve recovery in the future would be to carry out more repeat cultures to offset the losses associated with contamination.

Given the inevitable loses of viable *M. bovis* during samples processing, and the balance to be struck in all TB culture protocols between decontamination and the ability to recover the target *M. bovis* cells, there is a limit to the sensitivity that can be achieved. This is especially relevant when limited amounts of faecal samples are collected (2 g in the case of this study). Work in this study also showed that only about 5% of *M. bovis* was recovered from spiked samples which is likely to be typical for TB culture protocols but shows there is scope to improve the sensitivity of culture methods used.

In addition, it is also possible that the metabolic state of *M. bovis* present in faecal samples is not conducive to growth, and hence detection, using current mycobacterial culture media—so called viable but non‐culturable (VBNC) organisms. Members of the *Mycobacterium tuberculosis* complex are known to enter a dormant state in response to lack of nutrients/low oxygen levels (Caño‐Muñiz et al., 2017). It is therefore possible (though not established) that *M. bovis* may exist in such a state following transit through the cow gut and this may explain the results compared to spiking a laboratory maintained strain into faeces.

The use of PCR was an attempt to use a culture‐independent detection method which did not rely on successful growth of *M. bovis* in the laboratory. PCR detection would provide some evidence of shedding of *M. bovis* in the faeces of TB infected cattle. However, PCR will detect DNA from both alive and dead organisms, and hence does not provide definitive evidence of viable organisms and hence the potential infectiousness in cattle faeces. The real‐time PCR assays targeted the RD4 region (specific to *M. bovis*) and a universal bacterial assay to check the quality of DNA. The universal bacterial assay demonstrated that amplifiable DNA was reliably obtainable using the method, although there was some variation in the Ct values obtained (from C_T_ 25–35). This could be explained by variation in the amount of overall DNA or different levels of residual PCR inhibitors. However, dilution and subsequent PCR amplification of a selection of DNA samples (data not shown) did not result in improved C_T_ values suggesting inhibition was not a significant issue. However, it is possible that some inhibition was occurring in some samples given the known challenges of testing faecal samples by PCR.

DNA extraction from the culture inoculum (rather than directly from faeces) as part of the PCR testing process unfortunately did not result in a more sensitive means of detecting *M. bovis* in faecal samples as had been hoped. The limit of detection was similar to a highly optimized PCR for direct detection of *M. bovis* from badger faeces (Travis et al., 2011), where the limit of detection was also approximately 10^5^ *M. bovis*/g faecesThese results illustrate the challenges of conducting PCR analysis on faecal samples and suggest that the culture‐based methods used in this study were a more reliable way of detecting *M. bovis* in faecal samples. Culture‐based methods also detect viable *M. bovis* rather than *M. bovis*‐derived DNA which is a more meaningful measure in the context of potential disease transmission routes.

However, others have reported more sensitive assays (10^3^ *M. bovis* per gram faeces) for detection of *M. bovis* in badger faeces (King et al., 2015) and PCR assays for detection of MAP from bovine faeces have reported limits of detection as low as 10 organisms per gram faeces (Sevilla et al., 2014). Therefore, it is possible that further development and optimization of a PCR based method could improve the limits of detection and prove a more useful tool for detection of *M. bovis* in cattle faecal samples. Possible approaches could be direct DNA extraction from faeces (as opposed to extraction from culture inoculum used in this study), alternative DNA extraction methods and kits designed specifically for faecal samples or avoiding the heat inactivation step employed in this study which may have reduced sensitivity (heat inactivation was employed in this study to allow containment level 3 samples to be deactivated to allow subsequent PCR analysis in containment level 2 laboratories).

This work was an initial study to investigate the frequency and bacterial load of *M. bovis* in the faeces of naturally and experimentally TB infected cattle and its limitations have been discussed. Future work could build on this study to further refine our understanding of the potential risk of spread of TB via slurry and manure. This could involve more intensive sampling and testing of faecal samples from a larger number and different classes of cattle (including non‐visible lesioned TB reactors), development of sampling and testing regimes to improve the limit of detection and targeted sampling of premises (individual cattle and environmental samples) suspected of having high levels of shedding or issues with disease persistence due to potential environmental contamination (Fielding et al., 2020).

In conclusion, the results of this study show that the frequency of *M. bovis* shedding in the faeces of naturally and experimentally infected cattle from the UK and Ireland (including VL and non‐VL animals) is extremely low. The results suggest that the risk of subsequently spreading TB through slurry or manure use on agricultural land may be lower than suggested in some of the historical literature. The results reported here could be used to inform any reconsideration of current risk assessments and guidelines on the disposal of cattle manure and slurry on agricultural land.

## AUTHOR CONTRIBUTION

Si Palmer (carried out sample collection, laboratory work and result analysis), Gareth A Williams (carried out laboratory work and result analysis), Colm Brady (collected and supplied samples from Irish TB reactors), Eoin Ryan (supplied and coordinated the analysis of samples from Irish TB reactors), Colm Brady (collected and supplied samples from Irish TB reactors), Karolina Malczewska (inspected TB reactors as Official Veterinarian from UK slaughter house and assigned animals as visible lesioned), Tim J Bull (guided TiKa method work, carried out limit of detection work for the TiKa method), Philip J. Hogarth (helped conceive study) and Jason Sawyer (designed study, carried out PCR analysis, main author of manuscript). All authors reviewed the manuscript.

## CONFLICT OF INTEREST

Tim Bull is a director of TiKa Diagnostics Ltd.

## Data Availability

The data that support the findings of this study are available from the corresponding author upon reasonable request.
